# Effects of Mock Facebook Workday Comments on Public Perception of Professional Credibility: A Field Study in Canada

**DOI:** 10.2196/12024

**Published:** 2019-04-18

**Authors:** Cynthia Weijs, Jason Coe, Serge Desmarais, Shannon Majowicz, Andria Jones-Bitton

**Affiliations:** 1 Department of Community Health Sciences Cumming School of Medicine University of Calgary Calgary, AB Canada; 2 Department of Population Medicine Ontario Veterinary College University of Guelph Guelph, ON Canada; 3 Department of Psychology University of Guelph Guelph, ON Canada; 4 Faculty of Applied Health Sciences School of Public Health and Health Systems University of Waterloo Waterloo, ON Canada

**Keywords:** social media, professionalism, trust, professional-patient relations, medical education

## Abstract

**Background:**

There is considerable discussion of risks to health professionals’ reputations and employment from personal social media use, though its impacts on professional credibility and the health professional-client relationship are unknown.

**Objective:**

The aim of this study was to test the extent to which workday comments posted to health professionals’ personal Facebook profiles influence their credibility and affect the professional-client relationship.

**Methods:**

In a controlled field study, participants (members of the public) reviewed randomly assigned mock Facebook profiles of health professionals. The 2×2×2 factorial design of mock profiles included gender (female/male), health profession (physician/veterinarian), and workday comment type (evident frustration/ambiguous). Participants then rated the profile owner’s credibility on a visual analog scale. An analysis of variance test compared ratings. Mediation analyses tested the importance of credibility ratings on participants’ willingness to become a client of the mock health professional.

**Results:**

Participants (N=357) rated health professionals whose personal Facebook profile showed a comment with evident frustration rather than an ambiguous workday comment as less credible (*P*<.001; mean difference 11.18 [SE 1.28]; 95% CI 8.66 to 13.70). Furthermore, participants indicated they were less likely to become clients of the former when they considered credibility (standardized beta=.69; *P*<.001). Credibility explained 86% of the variation in the relationship between the type of workday comment and the participant’s willingness to become a client of the health professional.

**Conclusions:**

This study provides the first evidence of the impact of health professionals’ personal online disclosures on credibility and the health relationship. Public perceptions about professionalism and credibility are integral to developing the evidence base for e-professionalism guidelines and encouraging best practices in social media use.

## Introduction

### Changing Context of Professionalism

Professionalism is important to the health professional-client relationship (hereafter, the health relationship) [1]. Perceptions of professional credibility, generally defined as caring, competence, and trustworthiness [2], underpin clients’ sense of professionalism [1,3]. Typically, such impressions are formed during health interactions, though widespread internet use has introduced new contexts for impression formation. In one survey, 80% of US internet users sought health information online, with 44% specifically searching for information about health professionals [4]. Social media have garnered attention as being a risk to health professionals who use such sites in their personal lives. One site, Facebook, may reveal significant personal information that health professionals would be unlikely to share in the course of a client interaction. Given Facebook’s immense popularity with the public [5,6] and health professionals [7-11], and the call for evidence about public impressions of credibility developed in this new context [12], we explored public impressions of health professionals who use Facebook in their private lives.

### Reputation Risk When Private and Professional Lives Mix Online

An important issue is the need to strike a balance between health professionals’ right to use Facebook as private citizens and their duty to protect professional reputation and health relationships [13,14]. Reputation damage, as evidenced by employment loss and regulatory discipline [15,16], has occurred. However, the consequence to the health relationship from health professionals’ personal use of Facebook, or whether patients notice these behaviors or see them as inappropriate, is complex and less evidenced. Yet, 49 out of 72 (68%) of medical students surveyed in a social media and professionalism intervention reported the mixing of their personal and professional lives on social media sites as a key concern [17].

Normative beliefs about online or e-professionalism among some practicing health professionals and trainees differ considerably [7-9,11,18], perhaps mirroring the professionalism transition seen in the academic and medical education literature, from the old (unquestioned authority, paternalistic, and emotionally distant) to the new (client-centered, collaborative, and emotionally present) [19]. Some see e-professionalism as *a matter of taste*, overly rigid, individual, and impossible to attain [10]. Although it has been a decade since the advent of social media, very little evidence exists to inform social media guidelines about the interplay between personal online disclosures and professional trust and credibility [12] or whether expectations of e-professionalism might differ between health professional groups.

### Objectives

We explored the impact of workday comments that blur the line between health professionals’ private and professional lives as they are common and thought to have an impact on perceived professionalism [18,20-22]. We aimed to test (1) whether members of the public independently notice workday comments on Facebook profiles belonging to physicians or veterinarians, (2) whether such comments influence perceptions of professionalism (operationalized as credibility evaluations), and (3) the impact on the willingness of participants to engage with those health professionals (ie, become a client).

## Methods

### Informed Consent and Recruitment

The study was approved by the Research Ethics Board at the University of Guelph. Informed consent consisted of a welcome message and a letter of information on the landing page of a Web-based questionnaire. The sample was a convenience sample. Participants were recruited in person in communities across Southwestern Ontario or online, through local social media (ie, advertisements about the study on Facebook and Kijiji, a popular online classifieds site in Canada). Members of the public were approached in public spaces (eg, farmers’ markets, public trailheads, parks, soccer fields, and sports games) to take part in a study described as aiming to understand people’s first impressions of personality from online content. Participants who showed interest in participating were given a card with the researchers’ contact information and URL for the survey. The survey was open and voluntary. To control potential social desirability bias, no mention was made of health professionals [23]. Participants were encouraged to share the online survey link through email and Facebook. Data were captured automatically by a Web survey application, Fluidsurvey, and downloaded onto password protected and encrypted computers. After completing the survey, participants submitted their email contact information on a separate webpage to have a 1 in 350 chance of winning an Apple iPad.

### Survey Design

The first of 3 survey sections presented a randomly assigned mock Facebook profile in PDF format for participants’ review (a mock profile is available to readers from the authors upon request). The second survey section presented an adapted credibility scale on which participants rated the mock profile owner [2]. Finally, 16 demographic questions and 1 question asking participants about their willingness to become a client of the profile owner (a physician or veterinarian) were presented.

**Table 1:** Adapted credibility scale showing paired anchor characteristics for competence, caring, and trustworthiness (Cronbach alpha for the scale=.88). Two changes were made to the original validated scale^a,b^.

Competence	Caring	Trustworthiness
Novice/Expert	Insensitive/Sensitive	Phoney/Genuine
Unintelligent/Intelligent	Not understanding/Understanding	Unethical/Ethical
Incompetent/Competent	Self-centered/Unselfish	Untrustworthy/Trustworthy
Uninformed/Informed	Unconcerned/Concerned	Dishonest/Honest
Dim^a^/Bright	Uncaring/Caring	Immoral/Moral
Untrained/Trained	Doesn’t care about me/Cares about me^b^	Dishonourable/Honourable

^a^*Dim* replaced the original adjective, *Stupid*.

^b^This pair was removed from the scale owing to irrelevance to our study scenarios.

Profiles were made to simulate a real Facebook profile, although without working links and with names and headshot photos contributed by members of the research team with permission. Profiles were created using a 2×2×2 full factorial design, with the following attributes: mock profile owner gender (female/male), health discipline (physician/veterinarian), and workday comment type (evident frustration/ambiguous). Profile comments (ie, status updates) were modeled after actual status updates from previous studies of health professionals’ personal use of Facebook to authentically portray workday comments as they may be encountered online [18,20-22]. Profile photos of Sarah or Chad, the mock profile owners, were of a sunset, to control for potential bias related to facial features upon first impression. Several workday comments were pilot tested within the research team and with members of the public for content validity. In our post pilot test debrief, we asked participants what they understood the paired adjectives to mean. They all identified at least one of the exact labels of the scale (competence, caring, trustworthiness, or credibility) or a synonym of the scale (eg, professionalism). Additionally, we inquired about their views on the piloted comments as we aimed for a moderate comment in terms of emotional valence. On the basis of their feedback, we chose the following evident workday frustration comment:

What is it with some people?? I know I only went through 9 years of university...but really, I know what I’m talking about...yeesh!!!

Sarah or Chad

We also chose the following ambiguous workday comment:

Started with new electronic patient charts today...interesting experience for sure.

Sarah or Chad

Each participant reviewed the content of 1 randomly assigned mock Facebook profile without time constraints. On the next page, they completed the 17 personality ratings, an adapted credibility scale which included 3 subscales: competence (6 items), caring (5 items), and trustworthiness (6 items; Table 1) [2]. The scale originates from a widely cited study by McCroskey and Teven that validated a credibility scale composed of 3 related dimensions—goodwill (caring), competence, and trustworthiness [2]. We modified 2 items in the scale, replacing the original adjective, stupid, with dim in the competence subscale and removing 1 set of adjectives that was not relevant to our scenarios in the goodwill or caring subscale (Table 1).

Each item was represented on a visual analog scale, anchored at each end with an adjective describing less (eg, novice) or more (eg, expert) of the trait being assessed (eg, competence). Consistent with first impression research, participants were asked to move fairly quickly through all randomly presented rating scales, sliding the cursor to the place on the line between the 2 adjectives that they felt best represented the profile owner [24,25]. The question assessing a participant’s willingness to become a client of the mock profile owner was similarly represented, anchored by very unlikely and very likely.

Numeric gradations for all visual analog scales (0 to 100) were hidden to access participants’ immediate impressions. Only one response was possible, and all items had a nonresponse option of nonmovement from the middle position on the scale (neutral). Respondents were able to review and change their answers before moving to the next page and could not move backward in the survey so as to ensure collection of their *first* impressions. Duplicate entries were identified through a combination of internet protocol address and demographic details. All submitted surveys were included in the analysis, except those that were less than half complete or had fewer than 2 credibility subscales completed. The survey was pretested on a group of 10 individuals known to the researchers who were debriefed upon completing the survey about usability, clarity, functionality, and content validity of the credibility scale.

### Statistical Analysis

Univariable linear regression identified unconditional associations between the outcome variable (credibility rating) and covariate or control variables including the following: participant is a Facebook profile owner (no; yes); recruitment (in person; online); participant age (years); participant gender (female; male); annual household income before taxes (low ≤Can $ 74,999; high ≥Can $ 75,000); and participant’s profile viewing and rating times (in seconds). Covariates having a *P* value <.20 with the outcome variable were included in an omnibus analysis of variance (ANOVA), along with the 3 manipulated mock profile factors (profile owner gender, health discipline, and workday comment type), to determine credibility differences based on comment type.

Sequential linear regression determined the extent to which impressions gleaned from the online content of a personal Facebook profile impact the health relationship [26]. Specifically, credibility was assessed for its role as a mediator (intervening variable) in the relationship between profile workday comment type and the participant’s willingness to become a client of the profile owner. As above, univariable linear regression was employed to reduce variables eligible for inclusion in the model. In Step 1, the 3 manipulated variables in our study and any eligible covariates were entered into the model. In Step 2, credibility was added to the model. Mediation was supported if the intervening variable maintained a significant standardized beta coefficient when all other covariates were controlled [26]. Full mediation as per Tabachnick and Fidell was considered to have occurred when beta coefficients for significant variables in Step 1 were no longer significant in Step 2 [26]. Effect size, as per Cohen, and 95% CIs for the sequential regression model were calculated [27,28]. All statistical analyses were carried out in SPSS v23 (SPSS, Inc) using a significance level of *P*<.05.

## Results

### User Statistics

The total number of people invited to participate in the survey is unknown. A total of 6 surveys were removed owing to insufficient responses, leaving 357 usable surveys. Not all participants answered all questions, thus samples vary as noted below. A missing value analysis revealed that values were missing completely at random [26]. Participants were, on average, 40 years old (median 40 (SD 12.4); range 18-73) and 226 out of 355 (63.7%) were female. Participants’ income and education are shown in Table 2. Most participants (289/348, 83.0%) reported having a personal Facebook profile, and among those, 159 out of 285 (55.8%) reported checking it daily or several days per week (57/285, 20.0%). On average, participants viewed the mock Facebook profiles for 87 seconds (median 74 (SD 62); range 6.23-592 seconds), took 124.00 seconds (median 110 (SD 61.5); range 32-652 seconds) to rate the mock profile owners, and took 7.15 min (median 6.60 (SD 2.54); range 2.18-17.53 min) to complete the entire survey.

### Differences in Credibility Ratings of Health Professionals Having Either Evident Frustration or Ambiguous Comments on Mock Facebook Profiles

The following variables were included in the omnibus ANOVA: workday comment type, mock profile owner discipline and gender, participant age and gender, participant profile viewing time, and recruitment venue (in person, online). The ANOVA results yielded only one statistically significant main effect—workday comment type, *F*_1,339_=64.03; *P*<.001—that is, mock Facebook profile owners with an evident workday frustration comment were rated as significantly less credible than were those with an ambiguous workday comment (mean difference 11.2 (SE 1.3); 95% CI 8.66 to 13.70). The average credibility rating for evident profiles was 56.7 out of 100, whereas for ambiguous profiles it was 67.9 out of 100. The model-adjusted R^2^ was 0.188, indicating 19% of the variation in credibility ratings was due to the workday comment type.

### Understanding the Real-World Effect of Workday Comments and Credibility Ratings on Health Relationships

Sequential regression supported mediation, meaning that the effect of workday comment type (evident frustration versus ambiguous) on participants’ willingness to become a client of health professional profile owners was mediated by credibility ratings (Tables 3 and 4). Cohen f^2^ effect size for the model was large (0.86; 95% CI for f^2^ 0.62 to 1.18), meaning 86% of the variation in participants’ willingness to become a client of mock health professional profile owners was due to the addition of credibility rating to the model. Participants perceived the profile comment as a reflection of credibility, which subsequently impacted their willingness to become a client of the profile owner (higher credibility ratings associated with more willingness).

**Table 2:** Number (n) and proportion (%) of study participants within pretax annual household income and completed education categories.

Demographic characteristic		n (%)
**Pretax annual household income in Can $**		**n=335**
	<$30,000	50 (15)
	$30,000-$49,999	42 (13)
	$50,000-$74,999	75 (22)
	$75,000-$99,999	71 (21)
	≥$100,000	97 (29)
**Level of completed education**		**n=357**
	Completed or have some high school	52 (14)
	College diploma	66 (18)
	University degree	118 (33)
	Professional degree	48 (13)
	Graduate degree	72 (22)

**Table 3:** Step 1 of the sequential regression examining credibility as a mediator in the relationship between workday comment type and willingness to become a patient of the health professional for 318 study participants (*R*^2^=0.19; adjusted *R*^2^=0.17; and *F*_7, 310_=10.44).

Variable	Statistics	
	Standardized beta coefficient	*P* value
Participant age	−.03	.51
Participant income (ref^a^ ≤Can $ 74,999)	−.08	.17
Mock profile owner gender (ref female)	−.10	.05
Health discipline (ref physician)	.65	.21
Ambiguous workday comment (ref evident frustration)	.39	<.001
Online recruitment (ref in person)	−.02	.68
Scenario view time (in seconds)	−.08	.12
Credibility rating	—^b^	—

^a^Ref: referent.

^b^Variable not included in the model in Step 1.

**Table 4:** Step 2 of the sequential regression examining credibility as a mediator in the relationship between workday comment type and willingness to become a patient of a physician or client of a veterinarian for 318 study participants (*R*^2^=0.57; adjusted *R*^2^=0.56; change in *R*^2^=0.38; and *F*_1,309_=267.48).

Variable	Statistics	
	Standardized beta coefficient	*P* value
Participant age	−.02	.70
Participant income (ref^a^ ≤Can $74,999)	−.05	.18
Mock profile owner gender (ref female)	−.09	.02
Health discipline (ref physician)	.06	.11
Ambiguous workday comment (ref evident frustration)	.10	.03
Online recruitment (ref in person)	.06	.10
Scenario view time (in seconds)	−.06	.12
Credibility rating	.69	<.001

^a^Ref: referent.

## Discussion

### Principal Findings

Facebook functions as a space to share information and access social support [29,30], including sharing workday frustrations [31]. Health professionals, regulators, and educators aim to better understand health professionals’ personal use of social media and networking sites, especially Facebook, given its ubiquity in the personal lives of the general public and of health professionals [5-11]. This field study provides some of the first evidence that the blurring between private and professional lives that is inherent on health professionals’ personal Facebook profiles warrants the attention of health professional communities. Participants independently noticed a subtle workday frustration comment on a mock health professional’s personal Facebook account and perceived that individual as less credible than a mock health professional with an ambiguous workday comment. Participants also indicated less willingness to engage professionally with the profile owners who posted evident versus ambiguous frustration comments.

We found a small, though not statistically significant, difference between participant credibility ratings of physicians and veterinarians. Given the different health contexts for physicians and veterinarians, the findings of this study suggest that the public has expectations of online professionalism that warrant further exploration across a range of health professions to broaden our understanding of credibility evaluations in relation to and beyond those studied here.

### Comparison With Previous Work

Our results seem consistent with a conventional view of professionalism and suggest that current cautions around health professionals’ personal use of Facebook are not unreasonable [32]. Yet, mock profile owners in our study were rated somewhat less harshly (ie, still rated above the midpoint of the professionalism scale at 57 out of 100) than those in a similar study [33]; however, study design features may account for this difference. Jain et al had employees of a US university rate appropriateness (their measure of professionalism) of workday comments about patients made by medical students on a Likert scale (from 1-very inappropriate to 5-very appropriate); the average score was 1.88 [33]. Participants in that study were informed that the content was publicly available and searchable on Facebook and were asked to rate behaviors that were circled in red. In addition, workplace comments were combined for analysis, blending milder and more severe comments, all of which may have contributed to harsher ratings [33]. In contrast, we aimed to control socially desirable responses by having participants review the profile for as long as they wished, leaving them to independently notice both the workday comment and that the individual was a health professional.

In another study, Clyde et al suggest that clients may be motivated to search for health professionals online to supplement information available on a workplace website [34]. They found that Facebook profiles belonging to mock physicians with healthy personal information (eg, reading and hiking) were perceived as significantly more professional than profiles that limited content solely to information about mock physicians’ education and current practice. Clyde et al interpreted this to mean that access to personal information allowed participants to better judge physicians’ professionalism attributes [34]. Their finding was unexpected [34], perhaps because conventionally, personal self-disclosure (even of a positive nature) by physicians is thought to lower professional boundaries and is largely perceived as having a detrimental effect on the health relationship [35,36]. However, as with this study, their findings highlight the importance that health professionals’ personal disclosures online may have with patients.

### Strengths and Limitations

Key strengths of this field study were the random presentation of mock profiles and the realistic context of the survey design, which parallels the way a health professional’s Facebook information may be discovered online. The use of indirect questions to assess judgments of health professionals [37] and anonymity in survey completion [38] likely limited social desirability responses. The proportion of females (62%) to males in this study was more representative than in traditional survey methods, where women often comprise 71% to 77% of the participants [39], and the average age of participants in this study (40 years) is similar to the average age of a social media user (38 years) [29].

Nevertheless, choosing a health care provider involves more factors than were measured in this study, such as location and word-of-mouth referrals. Facial gestures and physical traits also influence impressions [40], as do characteristics of the trustor [41], comments posted by friends, and personality traits of the trustee [42,43], which were not examined here.

### Implications for Practice and Social Media Guidelines

In a recent exploration of dentists’ and patients’ attitudes toward social media use, 36% of patients reported searching for their dentist or doctor on social media. Other findings highlighted patients’ desire for more information about their health professionals (eg, by reading online reviews and qualifications) [44]. These findings underscore the near impossibility for health professionals in defending strict boundaries between their private and professional selves online but, rather, suggest the value of further research to better understand the impacts of unintentional *patient communication* of this type.

We conclude that health professionals, especially early-career professionals who have yet to build their professional reputation, should be mindful of their credibility with the public when using Facebook in their private lives. Adhering to social media guidelines that take a traditional or conventional view of professionalism is a reasonable first step, especially around workday comments. Although confidentiality breeches are more serious and have been a focus of attention for regulators and educators, they are rare [12,18], whereas, workday comments are common on Facebook [12,18-20,22]. We acknowledge that such a recommendation is associated with significant burden for health professionals, who have a right to access social support on Facebook when acting as private citizens. However, these rights need to be balanced with their professional obligation to maintain positive health relationships [13].

### Conclusions

Although a fuller understanding of e-professionalism is ongoing in this field, given the profound effect of digital technologies on society, social media guidelines should incorporate early evidence to prevent credibility damage because (1) trust, once broken, is difficult to rebuild [45]; (2) Facebook is seemingly rooted in society, with 1 in 5 Americans using social media for health care decisions (94% of whom reported Facebook as their trusted source) [46]; and (3) recent evidence suggests that health professionals do indeed *friend* patients [44]. This study underscores that the blurring of professional and personal lives on social media is important to understand and manage. Better elucidating patients’ differing perspectives of e-professionalism around various workday comments and identifying contextual influences, such as views about whether Facebook is private or public, will better define expectations of e-professionalism, which may in turn alleviate some of the concerns and reticence among health professionals to participate in social media.

Furthermore, an untapped area of health communication research includes the evaluation of health professionals use of social media skills in the public domain, that is, when, as private citizens, they participate in broader public online discussion about health matters within their professional domain (eg, posting comments in the comments section in news articles about vaccination).

## Abbreviations

ANOVA: analysis of variance
